# Navigating the Complexities of Nursing Documentation When Patients Have Access to the Content: A Qualitative Study

**DOI:** 10.1111/jan.16502

**Published:** 2024-10-01

**Authors:** Birgitte Lerbæk, Kathrine Hoffmann Kusk, Lone Jørgensen, Britt Laugesen

**Affiliations:** ^1^ Unit for Psychiatric Research, Psychiatry—Aalborg University Hospital Aalborg Denmark; ^2^ Research Unit Thisted Aalborg University Hospital Thisted Denmark; ^3^ Clinical Nursing Research Unit Aalborg University Hospital Aalborg Denmark; ^4^ Department of Clinical Medicine Aalborg University Aalborg Denmark; ^5^ Centre for Clinical Guidelines, Aalborg University Aalborg Denmark

**Keywords:** documentation, focus groups, nurse–patient interaction, qualitative approaches

## Abstract

**Aim:**

To explore how Danish registered nurses (RNs) in hospitals experience documenting nursing care in electronic patient records when the content is accessible to patients.

**Methods:**

In a qualitative research design, data were generated in six focus groups conducted in late 2022 and early 2023, comprising 31 RNs employed in inpatient wards at a university hospital in Denmark. Subsequently, qualitative content analysis was applied to the gathered data.

**Results:**

The findings include three themes: (1) weighing one's words, (2) building trust or triggering conflicts and (3) risking loss of knowledge. Together, these three themes illustrate the complexities that RNs navigate when patients have access to the content of nursing documentation.

**Conclusion:**

Patients' access to nursing documentation requires RNs to navigate a complex interplay of factors, including awareness of language‐use, influence on the nurse–patient–relative relationships, and the risk of losing essential knowledge. Therefore, although patients' access to nursing documentation can induce a positive change in terms of strengthening the professional focus on documentation, it can also result in changes in documentation practices in ways that may compromise nursing documentation as a working tool.

**Implications for the Profession and Patient Care:**

The findings emphasize an urgent need to explore and discuss how sensitive nursing observations can be shared in a safe and appropriate way when patients have access to the documentation. Furthermore, to prevent misunderstandings and conflicts with patients, it is essential to focus on and prioritize patient involvement in nursing documentation.

**Impact:**

RNs navigate complex practices when patients have direct online access to nursing documentation content. It is crucial to clarify which content nursing documentation should entail and how sensitive nursing observations can be shared in a safe and appropriate way.

**Reporting:**

The COREQ checklist was used for reporting.

## 1 Introduction

Keeping patient records is an integral and essential part of healthcare and treatment delivery that serves multiple purposes (Ministry of Health 2021a; Moriarty et al. 2019). One such purpose is to communicate information about nursing care, patient progress and outcomes. Documentation practices have significant implications for patient safety and continuity of care since keeping records is necessary for interprofessional information sharing, both within and across healthcare disciplines (Ministry of Health 2021a, 2021b; WHO 2007).

In recent years, there has been an increase in the documentation that registered nurses (RN)s are expected to manage, and there has been a move towards increased patient involvement and transparency in care and treatment (Bøgeskov and Grimshaw‐Aagaard 2019). Furthermore, nursing documentation contained in electronic patient records (EPRs) is increasingly shared with patients through online platforms (D'Costa, Kuhn, and Fritz 2020). These changes have implications for RNs documentation practices, and this study explored how Danish RNs experienced documenting nursing care in EPR when the content is accessible to patients.

## 2 Background

Nursing documentation in EPRs must reflect needs and care provision related to the individual care receiver. Common examples of nursing documentation include patient assessments related to care planning, implementation and evaluation of nursing interventions, vital signs, weight, height and documentation related to the administration of prescribed medication. Notably, healthcare authorities and local organisations are responsible for making decisions related to the format and policies for keeping EPRs. Currently, most clinical documentation is conducted using a computerised records system (Evans 2016).

The forms and practices related to nursing documentation have changed over time. Such changes have often been related to demands or regulations concerning nursing documentation or to changes made in the electronic systems used to store the records. In the Danish context, information related to consultations with doctors at hospitals has been accessible online to patients for years. However, a recent change has allowed nursing documentation contained in EPR to be shared with patients through online platforms. Notably, such changes aimed at increasing patient accessibility to health records have also been internationally implemented (Johansen et al. 2019; Zanaboni et al. 2020). In Finland, patient access to nursing documentation has been mandatory since 2016 (Essén et al. 2018). This move towards increasing patient accessibility to EPR is consistent with recent trends in contemporary healthcare settings, such as patient involvement and person‐centred care (Johansen et al. 2019; Zanaboni et al. 2020). Furthermore, patients have reported that online access to health record information in general is beneficial for keeping track of their treatment and preparing for appointments at the hospital (Zanaboni et al. 2020). These patients have also highlighted some of the clinical advantages of access to health record information, such as enhanced knowledge about their condition, improved self‐care, greater empowerment and easier communication with healthcare professionals (Zanaboni et al. 2020). Another advantage reported by healthcare professionals is increased communication with patients about the content of the EPR, through which they receive questions or comments related to missing information or mistakes (Johansen et al. 2019).

However, increased access to EPR may impact the documentation practices of RNs. In recent years, policies related to concepts such as patient participation have contributed to an increased focus on documenting *along with* the patient, as opposed to RNs producing documentation *about* the patient. Research has emphasised that the intentions to involve the patient in these practices are difficult to transform from policy into direct patient care (Kollerup, Vinther, and Jørgensen 2020). Although involving patients in nursing documentation may be assumed to improve documentation quality in terms of the content becoming summative and time accurate, this practice can be challenged by discrepancies between patients' expected needs (such as rest or a lack of resources) and RNs' needs (Kollerup, Vinther, and Jørgensen 2020). Furthermore, notable ethical dilemmas are embedded in nursing documentation practices, such as maintaining a balance between describing deviations in patients' health conditions and providing proof of nursing care to ensure professional accountability (Jørgensen and Kollerup 2022). Therefore, although producing nursing documentation along with the patient would increase the focus on patient preferences, the ethical dilemmas embedded in current documentation practices may become more profound.

Furthermore, existing knowledge on the potential influence of increasing patients' access to EPR is sparse, with only a few studies exploring healthcare professionals' perspectives in this context (Johansen et al. 2019; Zanaboni et al. 2020). A Norwegian survey identified minor differences in experiences and attitudes based on the different practices prevalent at different hospitals as well as between doctors and RNs (Johansen et al. 2019; Zanaboni et al. 2020). In this context, since nursing documentation has only recently become accessible to patients, there is a need to gain deeper insight into RNs' experiences and their descriptions of its influence on documentation practices.

## 3 Aim

The aim of the study was to explore how Danish RNs in hospitals experienced documenting nursing care in EPRs when the content is accessible to patients.

## 4 Methods

### 4.1 Design

This study adopted a qualitative research design, using focus groups for data generation. The study builds on the theoretical perspective of social constructionism. Within this perspective, knowledge about the surrounding world is understood as being continuously produced and shaped through human engagement in social interactions. In other words, individuals engage in the co‐construction of different versions of reality (Burr 2003). In this study, data were produced in participants' discussions in focus group sessions and is based on the interactional processes by which they presented their experiences and negotiated the expectations and attitudes towards the issue being studied.

### 4.2 Study Context

This study was conducted at a university hospital located in the North Denmark Region. In Denmark, nursing documentation is subjected to specific governmental standards and legislation that outline its purposes and mandatory content (Ministry of Health 2021a). These standards state that nursing documentation is primarily a working tool for RNs and a prerequisite for safe and continuous care and treatment. In addition, this documentation is considered an important source of information for patients, as it offers them the opportunity to draw insights and increase their involvement in their own care and treatment (Ministry of Health 2021a).

The patients' right of access to information from the EPR has been an integral part of patients' overall legal status in Denmark for years. Previously, accessing the information contained in EPR required Danish patients to first seek permission. However, with time, they were allowed to read selected notes made by hospital consultants, which they could access through a personal and secured online version of the EPR called the e‐journal. The e‐journal includes notes, diagnoses, treatments and discharge summaries beginning in 2009.

In recent years, patients' access to information on hospital‐based care and treatment has increased. In the North Denmark Region, since spring 2022, patients can also access nursing documentation. This information can be accessed through a personal and secure log in to the national homepage system Sundhed.dk (translates to Health.dk), which is administered by municipal and regional authorities and the Ministry of the Interior and Health (Danish Regions 2024; Jensen and Thorseng 2017). The accessible elements are listed in Table 1. In this system, the full name of the RNs who conduct the documentation is visible to the patients.

**TABLE 1:** Overview of elements accessible to patients online.

Elements from the EPR	Overview page Notes and epicrisis report Laboratory test results Image descriptions Cave Home measurements Medication overview Vaccinations Appointments Identity card (contact information) Plans and actions

### 4.3 Recruitment

Using purposeful sampling (Palinkas et al. 2015), 31 RNs were recruited to participate in six focus groups. Eligible participants were RNs working in inpatient wards at the university hospital. RNs who were a part of the hospital nursing documentation committee acted as gatekeepers in the recruitment process and invited colleagues from the hospital wards. Eligible participants were contacted by email, which included information about the study. They were then asked to contact the authors to make arrangements about their participation in the focus groups.

### 4.4 Focus Groups

Six focus groups (Barbour 2007; Morgan 1997) were conducted in late 2022 and early 2023. All authors were experienced in facilitating focus groups and took part in conducting the focus groups, with each focus group moderated by a member of the research team, along with another member present as an observant. The moderator and observer both took part in facilitating and engaging all attendees during the focus groups. Furthermore, the observer made notes during the focus groups. These were used in discussions about the focus group sessions among the authors.

The focus group discussions were facilitated using questions pertaining to specific themes related to nurse documentation. These were developed based on existing knowledge (see, e.g. Jørgensen and Kollerup 2022; Kollerup, Vinther, and Jørgensen 2020) and information on nursing documentation in clinical practice (act and legal guide to keeping nursing records) (Ministry of Health 2021a, 2021b) (Table 2). The focus groups lasted between 48 and 79 min and were conducted in a meeting room at the hospital locations. The focus groups were audiorecorded and transcribed verbatim.

**TABLE 2:** Themes and questions.

Theme	Research question	Facilitating question
Documentation practice	What characterises the participants' documentation practice?	Could you start by sharing something about how you do documentation in your everyday work practices? What is the focus of your documentation? Where does it take place? How do you prioritise what to document?
Change in patients' access	What are your experiences with nursing documentation now that this has become accessible to the patients?	What are your thoughts on patients' increased access to the nursing documentation?
		What are your experiences related to this?
		What does this mean to your documentation practices?
Pros and cons	What characterises the experienced pros and cons related to patients' increased access to nursing documentation?	Which pros and cons do you see related to patients gaining access to nursing documentation?
		How does this affect your approach to documenting nursing care?
Dilemmas	What characterises the dilemmas that can arise when nursing documentation is accessible to patients?	Which dilemmas have you experienced related to the patients having access to nursing documentation? Are there things that cannot be documented? Which things are omitted? How is information shared if omitted from the nursing documentation?
Close		Does anyone have anything else they want to share?

### 4.5 Analysis

The data material was analysed using qualitative content analysis (Graneheim, Lindgren, and Lundman 2017; Graneheim and Lundman 2004), involving a back‐and‐forth movement between the entire text and parts of the text. Initially, all authors read every interview to gain a sense of the overall meaning conveyed in participants' accounts. Next, the transcripts were divided into meaning units representing parts of the text concerning the aim of the study. Following the approach described by Graneheim and Lundman (2004, 106), the meaning units were then condensed into descriptions of the manifest content (what was said). Next, the condensed meaning units were interpreted to capture the latent content (what was talked about). Subsequently, the condensed meaning units were formulated into codes that further constituted the themes (Table 3). The formulation of the codes and themes involved a low level of interpretation, ultimately offering a description that closely aligned with the data (Graneheim and Lundman 2004). To ensure credibility, all authors extracted and interpreted the meaning units in the texts independently and then discussed their interpretations until a consensus was reached (Lincoln and Guba 1985). This process finally led to the initial formulation of three themes. The first and last authors developed and wrote up these themes, after which the descriptions for each were discussed among all authors. At the end of this process, the themes were written up and structured, along with corresponding illustrative quotes from participants. Table 2 provides examples illustrating this analytical process.

**TABLE 3:** Illustrative examples of the content analysis.

Data extracts	Condensed meaning units Manifest content	Condensed meaning units Latent content	Codes	Themes
We think a great deal about the language. You know, what words am I using now? And it might also be healthy enough that you're forced to reflect on what kind of language you use sometimes when you refer to the patients in writing (Registered nurse 3, Focus group 3)	The RNs think more about what language they use in the documentation. It is a good thing to do when you write something that the patient can access	The RNs experience a need to think more about what they write and how	What to write and how	Weighing one's words
And then I just always involve the patient in what I do because there is that basic trust between the involved parties. It also means that you're not afraid to write something because you've already said it in words. It's not like you're writing something and then saying something else. There must be congruence between what you do and say (Registered nurse 4, Focus group 3)	Involving the patient and making sure there is congruency between what you say and what you do are element that can facilitate trust. It may lessen the potential worries related to patients accessing nursing documentation	Involving the patient in nursing documentation can build trust	Documentation as a trigger of conflict	Building trust or triggering conflicts
I have noticed that if there's something that I have observed or noticed, maybe without having a specific professional justification but more a subjective feeling, I might articulate it to the patient but refrain from documenting it because it could become stigmatizing. It could be, for example, patients admitted for detoxification (Registered nurse 2, Focus group 1)	Reasons for not documenting include holding back observations that are based on subjective feelings and wanting to avoid stigmatisation of patients	Professionally unjustified observations may be omitted from the documentation	Alternative documentation practices	Risking loss of knowledge

Throughout the analysis, the application of the social constructionist perspective was enacted by engaging with the data and developing interpretations (Burr 2003). These interactions with the data helped attain a detailed understanding of the realities presented in the participants' accounts. Engaging with the participants' accounts allowed us to combine these with some broader assumptions regarding the underlying meanings embedded within the actual talk and text.

### 4.6 Ethics

The study complied with the established ethical guidelines for health research (Ministry of Higher Education and Science 2014; World Medical Association 2022). The study was approved by ward managers at the university hospital, and the RNs participated voluntarily and signed an informed consent form after receiving written and oral information about the study. The consent form followed guidelines from the hospital research administration. Anonymity and confidentiality were ensured by coding data and exchanging ID numbers for the respondents' names. The regional research administration approved the study, and it is a part of the North Denmark Region's record of processing activities (Project ID: F2022‐113). Furthermore, the study followed the European Union's General Data Protection Regulation article 30, and data have been stored following rules and regulations of the EU‐data protection regulation (The European Parliament and The Council of the European Union 2016). According to current legislation and the Danish National Center for Ethics, health research based on qualitative data, such as those from focus groups, does not need ethical approval (Ministry of the Interior and Health 2020).

### 4.7 Rigour and Reflexivity

Establishing trustworthiness included a process of addressing the rigour criteria of credibility, transferability, dependability and confirmability as described by Lincoln and Guba (1985). Addressing these four criteria, we drew on source and investigator triangulation (credibility, dependability and confirmability). These aspects are elaborated in the description of data analysis and in the study limitations. We provided adequate and proper descriptions to support the readers' transferability judgements (transferability). As an example, we explicitly described the design, theoretical stance and how these were enacted in methods used for generating and analysing data. Finally, we kept an audit trail throughout the processes of data generation and analysis to extend confidence in how our findings could be confirmed by other researchers (confirmability).

Reflexivity is critical to rigour, and to address reflexivity and clarify the specific roles of the authors, this section provides information related to the positions, experiences, and contributions of each author in conducting this research (Barbour 2007). Adopting the social constructionist perspective in this study meant that all the authors had to play active roles in the co‐construction of the studied phenomenon. The authors did this by engaging themselves in the research process by interacting with both the participants and the data, thus unavoidably impacting the research.

All four authors were experienced qualitative researchers: three with a background in nursing and one in sociology. All authors are female. At the time of conducting this study, the four authors were engaged in research and were affiliated with the university hospital where the study was conducted. None of the authors had any collegial relations with the participating RNs. The first and last authors were involved in the local board for nursing documentation at the university hospital.

## 5 Findings

### 5.1 Participants

The RNs came from a variety of specialties and represented both medical and mental health care settings. Table 4 provides additional information on the participants and the focus groups.

**TABLE 4:** Characteristics of focus groups and participants.

Focus group	Participants (*n*)	Age Mean (range)	Years as RN Mean (range)	Years in ward Mean (range)	Hospital	Hospital departments	Duration
1	4	30 (24–38)	6 (1–12)	2 (1–6)	Medical	Emergency	0:47:51
						Medical	
						Surgical	
2	6	42 (29–52)	19 (5–29)	7 (0–22)	Medical	Medical	0:55:05
						Surgical	
						Oncology	
						Haematology	
3	5	53 (46–61)	30 (21–38)	9 (1–15)	Medical	Emergency	0:54:49
						Medical	
						Surgery	
						Intensive care	
4	4	52 (39–65)	29 (16–42)	11 (2–23)	Medical	Neurology	0:54:28
						Gynaecology	
						Oncology	
5	5	33 (24–57)	4 (1–10)	4 (1–10)	Mental health	Child and adolescent psychiatry	1:19:54
						Affective disorders	
						Psychosis	
						Intensive care	
						Emergency	
6	7	42 (26–63)	13 (2–31)	6 (0–17)	Mental health	Psychosis	1:17:36
						Affective disorders	
						Mixed diagnosis	
						Acute, intensive	
						Intensive	
Total	31	43 (24–65)	17 (1–42)	7 (0–22)			6:09:43

The content analysis led to three themes: (1) *Weighing one's words*; (2) *Building trust or triggering conflicts* and (3) *Risking loss of knowledge*, which illustrate the complexities that the RNs navigated due to their awareness of patients' being able to access the content of nursing documentation.

### 5.2 Weighing One's Words

This theme illustrates that the RNs' awareness of patients' access to nursing documentation through online platforms compelled many of them to approach nursing documentation differently. They described paying ‘*greater attention to what you write and how’* (Registered nurse 5, Focus group 3) and how they engaged in various practices of weighing their words:

We think a great deal about the language. You know, what words am I using now? And it might also be healthy enough that you're forced to reflect on what kind of language you use sometimes when you refer to the patients in writing. (Registered nurse 3, Focus group 3)

The RNs were more attentive to how they referred to patients when documenting nursing care. Furthermore, they increased their focus on the use of specific language, which was perceived as a welcome change to ensure that the language used was respectful. As part of weighing ones' words, some RNs expressed that they paid more attention to writing in non‐stigmatising and non‐judgemental language. One of the participants explained:

Well, it's much about writing ‘I see’, ‘I hear’, so, what I'm observing, but without interpreting anything from it. And, at our unit, it can also be something like: ‘Mom did not hear the child crying. Continued sleeping. Slept heavily’. So being descriptive rather than conclusive. Rather than saying that ‘I got worried, she did not hear her child’, instead to write what I see, hear, and experience and how she is reacting when I wake her up. (Registered nurse 1, Focus group 4)

As such, RNs tried to present their observations using objective formulations to avoid language that could be considered stigmatising by patients. Documenting nursing care using objective language emerged as a way for the RNs to leave out their subjective interpretations and conclusions. This approach stimulated some RNs to document their nursing with a stronger focus on elaborating on the patients' symptoms:

We also focus on the objective […] and on writing whether they have delusions or are hearing voices with the specific examples. In that way, we do the physicians the favor that they know what it is rather than just writing: “hearing voices”, because you easily jump to the interpretation. I think we all do that very, very quickly. So, that's something that we are very focused on and discuss in many different professional fora related to documentation. (Registered nurse 3, Focus group 5)

Writing objective nursing documentation also had a cross‐disciplinary perspective. As described here, providing physicians with more objective observations instead of interpretations was perceived as something that could benefit the physicians in their work. Furthermore, the changed approach to documentation facilitated reflections among the RNs on the importance of the content and how it could best reflect their nursing practice. However, the changed approach required some practice, which involved describing ‘*what it is that we, as RNs, can support the patient by doing, or what kind of challenges the patient is having*’ (Registered nurse 5, Focus group). Changing the existing way of practicing nursing documentation was described by some participants as an ongoing learning process, which also included unlearning the practice of documenting within the physicians' domains and increasing their focus on documenting more specific nursing care:

We've talked a lot about the lack of nursing‐specific documentation. We were very good at writing whatever the physicians' thought was important and what had been decided during rounds. We just weren't very good at describing how the patient was actually doing and what we had done […] So, it was with that as a starting point that we could say, ‘Now we need to stop, and we need to be more focused on what kind of nursing we're practicing’. (Registered nurse 3, Focus group 3)

Nursing documentation being accessible to patients seemed to induce reflections on nursing care and how to document patients' conditions and needs from a nursing care perspective, and not just from the physicians' perspective. This change in focus in favour of specific documentation related to nursing care was described by the RNs as important for the patients and as something that could help avoid double documentation. One said:

It has definitely impacted on the quantity of documentation in this transition, where we practice writing other things than we used to do. But when that's said, I do think it's nice that we can see that this is the reflections and observations of the nurse. We've also gained something—no doubt about that—or we're going to in the long run. We have another awareness of nursing‐specific documentation (Registered nurse 5, Focus group 3)

As described in the quote above, this changed approach facilitated the development of a stronger professional focus in RNs' documentation practice. However, some RNs expressed that the new approach to documentation challenged the notion of documentation being exclusively a working tool. In other words, nursing documentation became similar to writing *to* patients. Perceiving patients as additional recipients of the documentation meant that the language had to be adjusted accordingly. Nonetheless, most RNs continued to consider documentation as an important working tool where ‘*the patients have just been allowed to read along*’ (Registered nurse 3, Focus group 2). To these RNs, nursing documentation was, first and foremost, their working tool, and its primary objective was to convey patient‐related information to colleagues.

### 5.3 Building Trust or Triggering Conflicts

This theme unfolds how RNs navigate the complexities of nursing documentation, given that patients' and relatives' access can either build trust or trigger conflicts in the nurse‐patient‐relatives relationship.

Involving patients and relatives in the documentation process was perceived by the RNs as a key factor in building trust. The RNs reported adopting various approaches to achieve this:

It's not like I tell the patient, ‘Now, I'm writing this’. If I'm in the patients' room, I might ask, ‘Is it okay if I write it like this, or should I understand it in this way?’ just to make sure that I've understood it correctly and that I'm writing it in the right way. But otherwise, I don't do it, even though technically we should. (Registered nurse 1, Focus group 1)

Although the RNs felt that they had limited opportunities to directly involve patients in the documentation process, some attempted to align expectations by articulating the content of the documentation and asking the patients to validate the information narrated by them. In some cases, the RNs involved relatives to confirm information about the patients:

Sometimes, the patients aren't clear […], so it's often discussed with relatives or how they experience it. Some things are very technical and don't make sense to relatives, but they can, of course, read it. Then we may need to talk to them if they don't understand what we have written. (Registered nurse 3, Focus group 3)

In such cases, the RNs perceived that explaining the documentation was part of their role. However, they also reported challenges in involving patients and relatives in documentation, as their perspectives on the patient's situation did not always align. Consequently, disagreements could arise between RNs, relatives and patients regarding what and how to document. One of the participating RNs described:

Sometimes, it's complicated if we are not quite on the same page. Then I tell them, ‘I am going to write it this way, but I will also write your opinion’. (Registered nurse 2, Focus group 1)

In this context, the RNs agreed that documenting disagreements was difficult, time‐consuming and involved making compromises. Moreover, some of them revealed that they navigated potential disagreements by including both the RN and patient perspectives in the nursing documentation to maintain trust in the nurse‐patient relationship. Meanwhile, others perceived that reaching an agreement on the content of the documentation was essential.

And then I just always involve the patient in what I do because there is that basic trust between the involved parties. It also means that you're not afraid to write something because you've already said it in words. It's not like you're writing something and then saying something else. There must be congruence between what you do and say. (Registered nurse 4, Focus group 3)

This indicates that congruity in the RNs' conversations with patients and relatives and their documentation was considered imperative to building trust. Furthermore, the RNs perceived that it was easier to document sensitive aspects of nursing care if they involved the patients in the documentation process. However, while the RNs perceived that patients' access to documentation could help build a trusting relationship, they also agreed that some aspects of documentation could be potential triggers for conflict, as expressed in the following quote:

We just know that many of our patients have extremely poor self‐care. Describing that they haven't washed clothes and haven't taken a bath can also lead to conflicts. Or ‘what you're documenting is not true’ or ‘it shouldn't be in my medical record’. It's, of course, a taboo in the public and in the system that they can't manage it. But that [omission in documentation] also means that they don't get the right help on the other side. (Registered nurse 1, Focus group 5)

Therefore, documentation became a potential trigger for conflicts when it was closely related to tabooed topics, such as the lack of ability to self‐care, as depicted above. In such situations, the RNs had to carefully navigate the documentation of such aspects. On the one hand, the RNs knew that documenting such factors could trigger conflicts; on the other hand, this information and observations could contribute to the knowledge necessary for providing the best possible care and treatment to the patient. Specifically, in the case of mental health settings, the RNs reported that documentation could trigger aggressive or threatening behaviour in patients or relatives. As a result, the RNs had to find ways to protect themselves:

I had a patient on the phone who was threatening, really aggressive. Normally, I might have made a brief note about it, but I didn't because I didn't like the idea of my last name appearing so the patient could see who it was, they had spoken to. (Registered nurse 3, Focus group 2)

The core issue in such situations was concerned with the RNs' names appearing alongside the documentation they were responsible for, which compelled many of them to not always document the information they possessed about a patient. The participants shared various examples of unwanted contact initiated by patients, for example where colleagues had ‘*been asked out on a date*’ (Registered nurse 3, Focus group 3) or ‘*being called up by a patient*’ (Registered nurse 1, Focus group 3) after discharge. In such precarious situations, the RNs found contact by patients unacceptable. They stressed the need for anonymity to ensure their safety in the case of patients accessing nursing documentation.

### 5.4 Risking Loss of Knowledge

This theme shows RNs' perceptions of several factors that prevent them from documenting sensitive aspects of their patients' situations. This resulted in a dilemma where RNs had to choose whether to document, not document, or choose alternative ways of conveying information to colleagues.

Across all focus groups, the RNs reported an increased awareness of how subjective feelings or intuitions could be considered stigmatising if documented, as communicated in the following comment:

I have noticed that if there's something that I have observed or noticed, maybe without having a specific professional justification but more a subjective feeling, I might articulate it to the patient but refrain from documenting it because it could become stigmatizing. It could be, for example, patients admitted for detoxification. (Registered nurse 2, Focus group 1)

The RNs found it challenging to document concerns or observations for which they did not have established professional arguments. They were concerned that their initial intuitions, which they referred to as subjective concerns and observations, could be stigmatising, for example if they were related to the characteristics of certain vulnerable groups of patients. Therefore, instead of using documentation as a working tool to convey these observations, some RNs preferred to share such information verbally. However, this prevented the RNs from systematically involving their colleagues in the observations, in place of which they employed alternative ways of sharing knowledge with colleagues, as noted in the following quote:

Well, it's often about intuitions. Things you're not entirely sure about, which you could write freely in the old system where patients didn't read along. It can be many things. It can be when a mother says she wants to breastfeed, but everything about her seems to resist, and you think, ‘I could write that I have a sense that maybe it's not what she wants or that her body language says something else’. I wouldn't consider writing that today, knowing that the mother is reading along. It maybe something that I'll pass on orally to the evening shift. (Registered nurse 2, Focus group 4)

In some cases, patients' access to nursing documentation hindered the RNs from providing a comprehensive picture of the patients' situation in their documentation, since they chose to exclude their own interpretations and intuitions. These were elements of nursing care that the RNs would not hesitate to document previously. In addition, the RNs described that they chose not to report some initial points of attention regarding patient care and treatment if they included sensitive aspects of care, since they are difficult and time‐consuming to document. Instead, they conveyed such messages to their colleagues verbally:

I can just be afraid that it won't be documented, even when you really have to wrack your brain about how to write it, so the patient doesn't get upset. My colleagues understand what I write. I mean, I just refrain because I don't have time for this (Registered nurse 5, Focus group 2)

It seemed that the RNs felt insecure, and they did not possess sufficient knowledge on modifying the ways in which they usually documented aspects of nursing care that could potentially offend patients. As a result, they sometimes found it easier to simply not document certain details. In addition, the RNs expressed facing a dilemma when asked not to document sensitive information on the patients' situation shared by relatives:

There can be some things where you think ‘well, if we are to maintain this good relationship, then it shouldn't be documented’. Sometimes, the relatives call and tell me something they don't want to be documented. This can be about some intense things, like incest. So, there are some things that shouldn't be in the nursing documentation, but then it isn't the working tool we want it to be. There are definitely some dilemmas in that. (Registered nurse 6, Focus group 2)

The RNs also talked about the dilemma of possessing knowledge about which their patients were unaware as well as not being able to convey written information to colleagues. Overall, as a consequence of not documenting sensitive information, the RNs could not sufficiently involve their colleagues in the treatment process, which further presented the risk of compromising informational continuity. In some situations, RNs held back decisive knowledge crucial to the outcome of a pivotal decision that they could not document due to patients' access to nursing documentation. For instance, one RN narrated the following situation:

There is one of the parents who talk very inappropriately about their child in front of both the child and the staff to such an extent that we have considered whether the child should be removed from the home. Before this admission, this was a well‐functioning family. Therefore, we have waited to document until we have figured out what is going on. So, we use email correspondences as documentation and create it as drafts within the documentation system so that it is not accessible to the parents until we have figured out what is actually going on. (Registered nurse 2, Focus group 5)

If RNs shared such knowledge in their documentation, it would potentially compromise the objective assessment of the patient's situation since the parents would likely behave differently or withhold necessary information if they knew what they were being observed for. This would clearly compromise nursing documentation as a working tool. As a result, the RNs resorted to alternative ways of conveying and sharing information with colleagues, for example writing emails or post‐its.

From the descriptions above, it is evident that the RNs took recourse to alternative documentation methods when they perceived that the knowledge/information was important to share with colleagues but was not suitable for patients or relatives. Moreover, it seems that the dilemma of compromising nursing documentation as a working tool and risking the loss of knowledge was particularly prominent in situations involving difficult care and treatment decisions.

## 6 Discussion

This study aimed to explore how RNs in hospitals experienced documenting nursing care in EPRs when the related content was accessible to patients through online platforms. The findings illustrate the complexities that RNs have to navigate in view of patients' direct access to nursing documentation content. Patients' direct access resulted in changed documentation practices in relation to both language use, focus of the content and ways of translating nursing practices into documentation.

To the best of the authors' knowledge, this is the first study to explore RNs' experiences of documenting nursing care when patients have online access to the content. This may be due to differences in nursing documentation and patients' access to it worldwide. Traditionally, nursing documentation has been accessible to patients only to a limited extent. In the context of Denmark, until 2022, Danish patients could view their nursing documentation only if they applied for permission to view the same. However, recent years have been characterised by an increase in the sharing of medical information with patients, in terms of patients having increased online access to consultants' notes in their medical records (D'Costa, Kuhn, and Fritz 2020). With increased digitalisation facilitating this development, new opportunities and challenges related to RNs' documentation practices have emerged, as identified by the findings of this study.

Similar to the current study, a previously conducted systematic review revealed that multiple aspects must be accounted for when patients have access to healthcare documentation (D'Costa, Kuhn, and Fritz 2020). Furthermore, although patients' access to the information in their EPR has been described in the literature as having the potential to strengthen patient‐centred care, full access to written records has been found to be insufficient if not accompanied by additional oral explanation or summary (Bøgeskov and Grimshaw‐Aagaard 2019). This is consistent with the findings of the current study, where some RNs perceived explaining the documentation to patients and relatives as part of their role. Furthermore, patients' access to documentation serves to increase some RNs' focus on the importance of involving patients in the documentation process.

Increased information on care and treatment has the potential to empower patients and improve their trust in and knowledge of the care and treatment they receive (Bøgeskov and Grimshaw‐Aagaard 2019; Hägglund et al. 2022). Nevertheless, this study found that challenging and comprehensive changes are necessary in the existing documentation practice for patients to achieve the above‐mentioned benefits. Some RNs recognised that including patients' perspectives in the documentation was integral to maintaining or building trust in nurse‐patient relationships. However, in general, they found it difficult to directly involve patients in the documentation process because it was time‐consuming, indicating that an approach to documentation that is different than the usual practice is required (Kollerup, Vinther, and Jørgensen 2020). Previous research has also concluded that involving patients in the documentation process is a challenging task that is carried out only sparingly by RNs. Other challenges involved in patient participation include trying to meet the patients' expected needs and the RNs' needs in acute medical wards, despite the ideal physical location for carrying out such documentation being the patient's room (Kollerup, Vinther, and Jørgensen 2020). Nonetheless, the findings of this study underline the imperative of involving patients in documentation when they have access to the content contained in it. This is because patient involvement in nursing documentation may have the potential to prevent avoidable misunderstandings and potential conflicts while also enhancing nurse‐patient relationships.

Furthermore, this study found that patients' access to nursing documentation requires RNs to weigh their words, use objective language and leave out subjective interpretations and conclusions that could be considered stigmatising. This aligns with the findings of previous research (Hägglund et al. 2022), revealing that the language used by clinicians sometimes results in patients feeling judged or offended by what they read. This may be caused by errors, surprises, forms of labelling and the use of disrespectful wording in nurses' reports (Hägglund et al. 2022). The current study adds to the existing knowledge by emphasising that the awareness of patients' access to nursing documentation stimulates RNs to engage in various practices of weighing their words, such as writing in an objective language. Changing the documentation practice involved a learning process, which the RNs seemed positively engaged in. Moreover, this learning process facilitated a stronger professional focus in the documentation practice, which most of the RNs welcomed. In contrast, previous research reported a lack of confidence and motivation towards nursing documentation changes since it could complicate RNs' adherence to nursing documentation procedures (Bøgeskov and Grimshaw‐Aagaard 2019). Overall, it seems that nurses are divided between a positive view of documentation as something essential to their profession and a negative view of it being a meaningless burden that distracts them from their ‘real’ work, contradicts their professional identity, and does not benefit the patient (Bøgeskov and Grimshaw‐Aagaard 2019). According to the findings of this study, it seems that a modified documentation approach could facilitate discussions and considerations on what defines and entails nursing care and how it can be documented. This could be an effective way of ensuring that documentation continues to be a meaningful activity that not only ensures patient safety but also reflects nursing observations using common, objective, and professional language.

Although most RNs welcomed the changes in nursing documentation resulting from patients' access to the content, this study demonstrates that there may be multiple barriers to appropriate documentation when patients have online access to the content. These barriers included insecurity among RNs about weighing their words, the time‐consuming process of changing documentation practices and the risk of triggering conflicts with patients or relatives. In line with this study, previous research has underlined that RNs consider record‐keeping a complex activity owing to the variety of challenges they face, including lack of time to complete writing the records, increased patient admission and inadequate supervision of nursing documentation (Kamil, Rachmah, and Wardani 2019). Overall, this study highlights that patients' direct access to nursing documentation adds to the complex interplay of factors influencing nursing documentation since RNs have to consider sharing their observations with colleagues more carefully in such a context.

In the current study, it also seemed that the complexity of nursing documentation increased in situations in which the RNs perceived that patients' access to sensitive information could compromise clinical observations or trigger conflicts. Such situations could lead to the use of alternative approaches for nursing documentation, with some RNs refraining from documenting certain aspects of their nursing care—factors that could compromise patient safety. This result aligns with the findings of previous research, which demonstrated that clinicians maintain shadow records to prevent patients from potentially being harmed by the contents of their records (Balka 2010; Hägglund et al. 2022). The practices used for keeping ‘shadow records’, as observed in this study, included the use of post‐it notes and emails among colleagues and choosing only oral handovers. These alternative approaches were employed by the study respondents to ensure that sensitive information was shared only among colleagues.

According to current national guidelines and legislation, nursing documentation should include information about the current state of the patient as well as any potential problems (Ministry of Health 2021a, 2021b). The purpose of regulating nursing documentation is to ensure the availability of accurate and necessary documentation to ensure coherent and safe care and treatment trajectories of high quality (Ministry of Health 2021a, 2021b). In this context, the use of alternative approaches for sharing information among colleagues may present the potential consequences of risking loss of knowledge, breaching information continuity and compromising treatment. In this regard, the findings of this study emphasise the need to identify and discuss new ways in which sensitive nursing observations can be shared with colleagues in a safe and appropriate way when patients have access to such documentation. In addition, this study revealed that utilising alternative approaches to documentation or refraining from documenting is also linked to RNs wanting to protect themselves, since their names and surnames appear in the documentation accessed by patients. However, investigations into the issue of the anonymity of RNs and other healthcare professionals in healthcare documentation are lacking, even in the international literature. Notably, in the context of Denmark, de‐identified markers will be used to link documentation content to specific healthcare professionals from 2024, thus rendering them unidentifiable by patients reading the content.

### 6.1 Strengths and Limitations

The purpose of conducting focus groups was to explore the different meanings and ways in which perspectives are constructed in social interactions among participants. Following this method, this study included 31 RNs who participated in 6 focus groups. Drawing on the existing literature, this study aimed to include around 5 to 7 participants in each focus group. However, since the number of participants in each group varied, two of the focus groups comprised only four participants, which may have influenced the dynamic of these two groups. Nonetheless, the overall number of participants was deemed adequate (Barbour 2007).

Applying purposeful sampling, this study aimed to recruit RNs with experience in nursing documentation, representing great variations in terms of work experience across the medical and mental health specialties, as well as across different geographical settings of the university hospital. In practice, none were excluded from participating, and the recruitment strategy resulted in a quite heterogeneous group of participants, which is also reflected in the characteristics provided in Table 3. This contributed not only to a broader overview and more depth with regard to the perspectives presented by the groups but also lively discussions among the participants (Barbour 2007). Due to the heterogeneity of the group, we have chosen not to specify the participants further to ensure participant anonymity.

Furthermore, since this research was based on a single data generation method, researcher triangulation was applied throughout the research process to increase the credibility and validity of the study (Denzin 1978; Lincoln and Guba 1985). Therefore, by accounting for different perspectives on the topic being studied, researcher triangulation contributed to the richness and clarity of the research (Heale and Forbes 2013).

In addition, all the researchers who took part in conducting this study were experienced qualitative researchers, which contributed to the strength of this study in terms of addressing the complexities that emerged in the research process when researcher triangulation was applied (Johnson et al. 2017; Thurmond 2001).

### 6.2 Recommendations for Further Research

The overall finding underlines the necessity of conducting research focused on changes in the existing documentation practice. In this context, conducting participatory design studies involving RNs and other relevant stakeholders in a co‐design process for modifying the current practice could be useful. Furthermore, there is an urgent need to explore patients' experiences with direct access to nursing documentation.

### 6.3 Implications for Policy and Practice

The study findings emphasise an urgent need to explore and discuss the ways in which sensitive nursing observations can be shared with colleagues in a safe and appropriate way when patients have access to such documentation. Furthermore, this study highlights that focussing on and prioritising patient involvement in nursing documentation is essential to prevent misunderstandings and potential conflicts and to facilitate a healthy nurse‐patient relationship.

## 7 Conclusion

Documenting nursing care when the content is accessible to patients requires nurses to navigate a complex interplay of factors, including awareness of language use, the influence of nursing documentation on nurse‐patient‐relative relationships, and the risk of losing essential knowledge. Consequently, while patients' access to nursing documentation may induce a positive change in terms of strengthening professional focus on documentation, it may also change documentation practices in ways that could compromise the use of nursing documentation as a working tool.

## Author Contributions

All authors have agreed on the final version and meet both of the following criteria (recommended by the ICMJE):

1. substantial contributions to conception and design, acquisition of data or analysis and interpretation of data;
2. drafting the article or revising it critically for important intellectual content.

## Conflicts of Interest

The authors declare no conflicts of interest.

### Peer Review

The peer review history for this article is available at https://www.webofscience.com/api/gateway/wos/peer‐review/10.1111/jan.16502.

## Supporting information


Data S1.

## Data Availability

The authors have nothing to report.
